# Astroglial and microglial pathology in Down syndrome: Focus on Alzheimer's disease

**DOI:** 10.3389/fncel.2022.987212

**Published:** 2022-09-20

**Authors:** Octavio García, Lisi Flores-Aguilar

**Affiliations:** ^1^Facultad de Psicología, Unidad de Investigación en Psicobiología y Neurociencias, Universidad Nacional Autónoma de México, Ciudad de México, Mexico; ^2^Department of Pathology and Laboratory Medicine, University of California, Irvine, Irvine, CA, United States

**Keywords:** Down syndrome, Alzheimer's disease, astrocytes, microglia, β-amyloid, neuroinflammation, aging, cytokines

## Abstract

Down syndrome (DS) arises from the triplication of human chromosome 21 and is considered the most common genetic cause of intellectual disability. Glial cells, specifically astroglia and microglia, display pathological alterations that might contribute to DS neuropathological alterations. Further, in middle adulthood, people with DS develop clinical symptoms associated with premature aging and Alzheimer's disease (AD). Overexpression of the amyloid precursor protein (*APP*) gene, encoded on chromosome 21, leads to increased amyloid-β (Aβ) levels and subsequent formation of Aβ plaques in the brains of individuals with DS. Amyloid-β deposition might contribute to astroglial and microglial reactivity, leading to neurotoxic effects and elevated secretion of inflammatory mediators. This review discusses evidence of astroglial and microglial alterations that might be associated with the AD continuum in DS.

## Introduction

Down syndrome (DS) or trisomy 21 is the most common autosomal aneuploidy in human births, and it is the single most common genetic cause of intellectual disability (Antonarakis, 2017). Individuals with DS display different phenotypic characteristics, including craniofacial alterations, skeletal anomalies, hypotonia, congenital heart disease, gastrointestinal anomalies, seizures, sleep apnea, deficits in immune response, and leukemia (Antonarakis et al., 2020). Although these characteristics can lead to premature death, in recent years, people with DS have increased life expectancy, gradually approaching that of the general population (Glasson et al., 2016; Carr and Collins, 2018). However, upon reaching middle adulthood, people with DS commonly experience clinical symptoms associated with older age (Gensous et al., 2020), such as premature menopause, skin wrinkles, presbycusis, alopecia, premature graying of the hair, congestive heart failure, hypogonadism, hypothyroidism, osteoporosis atherosclerosis, diabetes, hypercholesterolemia, hypertension, and visual and auditory decline. In addition to the physiological manifestations of premature aging, people with DS present early cognitive impairment characterized by a decrease in memory skills (Godfrey and Lee, 2018), language skills (Vicari et al., 1994), social communication, motor skills, personal life, and community life skills (Hawkins et al., 2003). By the age of ~50 years, individuals with full triplication of chromosome 21 develop Alzheimer's disease (AD) dementia (Holland et al., 1998; Margallo-Lana et al., 2007; Zigman, 2013; Covelli et al., 2016; Fortea et al., 2021; Iulita et al., 2022) with an incidence of 88–100% in those older than 65 years (McCarron et al., 2017). The development of AD in DS could be associated with an overexpression of several genes located on chromosome 21, including the amyloid precursor protein (*APP)* gene, which encodes the amyloid-beta precursor protein (AβPP) (Wiseman et al., 2015), leading to an increase in Aβ production. Adults with DS over age 40 display overt AD neuropathology characterized by the deposition of extracellular amyloid plaques and the formation of neurofibrillary tangles (Wisniewski et al., 1985; Lemere et al., 1996; Mori et al., 2002; Davidson et al., 2018; Lott and Head, 2019).

*Postmortem* and *in vivo* studies in DS brains show pathological changes associated with AD, including brain atrophy (Mann and Esiri, 1989; Sadowski et al., 1999; Annus et al., 2016; Head et al., 2016), endosomal/lysosomal abnormalities (Cataldo et al., 2000), degeneration of cholinergic basal forebrain neurons, neurotrophin deregulation (Iulita et al., 2014), oxidative stress (Busciglio and Yanker, 1995), cerebral amyloid angiopathy (Carmona-Iragui et al., 2017; Head et al., 2017), white matter lesions (Lao et al., 2020), and neuroinflammation (Wilcock et al., 2015; Flores-Aguilar et al., 2020). Several studies have demonstrated the importance of inflammatory processes in AD pathogenesis (Liu et al., 2022), suggesting that Aβ could induce glial cell dysfunction (Uddin and Lim, 2022) and subsequent neurodegeneration. This review describes the pathological changes in astrocytes and microglia of individuals with DS and we discuss the implications of glial dysfunction in the AD continuum in DS.

## Astrocytes

Astrocytes constitute the largest glial cell population in the central nervous system (CNS). Astrocytes can be as heterogeneous as neurons at the genetic, physiological, and functional levels (Torres-Ceja and Olsen, 2022). Astrocytes play an essential role in the development and maintenance of the CNS. They provide structural support and participate in neuronal pathfinding and metabolism, ionic homeostasis, neurotransmitter release, and regulation of the blood-brain barrier (Allen, 2014). Astrocytes release trophic molecules, including thrombospondins (TSPs), cholesterol, nerve growth factor (NGF), brain-derived neurotrophic factor (BDNF), neurotrophins 3 and 4 (NT3, NT4), and tumor necrosis factor-α (TNF-α) (Clarke and Barres, 2013) involved in the modulation of synaptic transmission and synaptogenesis.

## Astrocytes in Down syndrome

Several studies have described neuropathological changes in the DS brain (Dierssen, 2012). In contrast, studies focusing on glial cell changes have received little attention. The number of glial cells, especially astrocytes, is altered during brain development in DS (Ponroy Bally and Murai, 2021). An increase in astrocytes and radial glial cells has been described in the frontal lobe of fetuses with DS at 18–20 weeks of gestation (Zdaniuk et al., 2011). Another study reported a lower expression of the glial fibrillary acidic protein (GFAP) in the hippocampus and temporal lobe of fetuses with DS only during the middle pregnancy period (Kanaumi et al., 2013). On the other hand, the fusiform gyrus and inferior temporal gyrus of fetuses with DS at 17–21 gestational weeks showed a higher percentage of GFAP-positive astrocytes (Guidi et al., 2018). Disruption of the interlaminar astroglial processes has been reported across the lifespan of individuals with DS (Colombo et al., 2005). A decrease in the number of astroglial interlaminar processes occurs within the first year of age, accompanied by an increase in immature astroglial cells in different regions of the DS brain (Colombo et al., 2005). Disruptions of the cortical astroglial architecture are more pronounced during adulthood. The absence of astroglial palisade has been reported in the dorsolateral region and the striate cortex of a 23-year-old individual with DS (Colombo et al., 2005). Degeneration of the interlaminar glial palisade progresses until adulthood, and instead, an increase in astrogliosis has been observed and associated with AD pathology in DS brains (Colombo et al., 2005).

In middle-aged and adults with DS (15–45 years old), there is a decrease in GFAP gene expression in the superior prefrontal cortex (Goodison et al., 1993). In contrast, GFAP protein expression increases after 30 years of age in the molecular layer of the hippocampus (Mito and Becker, 1993). In adults between the ages of 61 and 80, a partial reduction in the number of astrocytes and oligodendrocytes in the mediodorsal thalamic nucleus has been reported (Karlsen et al., 2014).

Such differential GFAP expression may be region specific and may change according to the progression of AD neuropathology.

In Ts1Cje mice, a mouse model of DS, the cerebellum displays a continuous increase of GFAP from youth to old age (Creaú et al., 2016), suggesting defective astrocyte production during aging. Some evidence indicates that GFAP expression is associated with increased micro-RNA-125b (miRNA-125b) levels in the temporal cortex of adults with DS between 63 and 73 years of age (Pogue et al., 2010). Micro RNAs (miRNAs) are post-transcriptional modulators of gene expression that regulate the stability and translation of their target messenger RNAs, suggesting that miRNA-125b contributes to astrogliosis during aging in DS.

On the other hand, several studies support the importance of the surrounding environment in DS neurological alterations. Nelson et al. (1997), used the DS (Ts16) mouse model to co-culture neurons and astrocytes, and demonstrated that wild-type neurons co-cultured with Ts16 astrocytes showed a decrease in choline acetyltransferase activity and a decrease in cholinergic neuron number, while Ts16 neurons co-cultured with wild-type astrocytes showed regular cholinergic activity (Nelson et al., 1997). In agreement with these results, studies have demonstrated that deficits of thrombospondin-1 (TSP-1, an extracellular matrix component involved in cell–cell and cell–matrix communication) in DS astrocytes can cause the alterations of dendritic spines and synapses similar to those reported in DS neurons (Garcia et al., 2010; Torres et al., 2018). In addition, astrocyte-conditioned cell media from DS astroglia causes toxicity to neurons and fails to promote neuronal ion channel maturation and synaptic formation (Chen et al., 2014). On the other hand, induced pluripotent stem cells (iPSCs) derived from individuals with DS show an increase in the number of astrocytes (Briggs et al., 2013). Down syndrome astrocytes differentiated from iPSCs exhibit high levels of GFAP, S-100β, and reactive oxygen species (Chen et al., 2014). Induced pluripotent stem cells-derived astrocytes negatively regulate secreted factors that can promote the formation of synapses, including TSP-1 and TSP-2, and alter the mTOR pathway in neurons (Araujo et al., 2017), suggesting a role of astrocytes in DS neuropathology.

## Ca^2+^ signaling in DS astrocytes

Hippocampal and cortical astrocytes obtained from Ts16 and Ts65Dn DS mouse models show impaired Ca^2+^ signaling. In the resting state, DS astrocytes show a higher concentration of cytoplasmic Ca^2+^ compared to euploid astrocytes (Bambrick et al., 1997; Muller et al., 1997). On the other hand, the inhibition of Ca^2+^ reservoirs in the endoplasmic reticulum induced a transient increase in cytoplasmic Ca^2+^ of 1,200 nM in Ts16 astrocytes compared to only 500 nM in euploid astrocytes (Bambrick et al., 1997). However, a stimulus-induced by serotonin or glutamate evoked transient Ca^2+^ increases from 400 to 600 nM in euploid astrocytes and from 20 to 150 nM in trisomic astrocytes, suggesting alterations in intracellular Ca^2+^ homeostasis in DS (Muller et al., 1997). Defects in Ca^2+^ homeostasis in astrocytes could affect cell proliferation and neuronal maturation (Bambrick et al., 2003). Finally, an increase in reactive astrogliosis has been described in basal ganglia calcification in DS (Takashima and Becker, 1985). Basal ganglia calcification may indicate premature aging (Mann, 1988).

## Oxidative stress in DS astrocytes

Altered mitochondrial activity and oxidative stress have long been associated with DS (Coskun and Busciglio, 2012). Several genes involved in the cell redox state are triplicated in DS, the most prominent being Cu^2+^/Zn^2+^ superoxide dismutase 1 (SOD1) (Pagano and Castello, 2012). Superoxide dismutase 1 scavenges reactive free radicals by catalyzing the dismutation of oxide anion into molecular oxygen and hydrogen peroxide. A high expression of SOD1 has been observed in the brains of people with AD between 59 and 97 years of age and in the temporal lobe of individuals with DS between 59 and 70 (Furuta et al., 1995). Mainly, SOD1 has been closely related to GFAP-positive cell processes (Furuta et al., 1995). Down syndrome brain tissue is susceptible to oxidative injury, mostly because increased SOD1 activity is not followed by an adaptive rise in hydrogen peroxide metabolism (Pagano and Castello, 2012). In neurons and astrocytes from human fetal DS cerebral cortical tissue, exposure to hydrogen peroxide shows that astrocytes are more resistant to oxidative damage (Sebastia et al., 2004). Down syndrome astrocytes show a lower basal content of superoxide ions and a higher clearance of hydrogen peroxide from the culture medium (Sebastia et al., 2004). In the presence of hydrogen peroxide, DS astrocytes maintain their concentration of intracellular superoxide and hydroperoxides at a lower level than normal astrocytes (Sebastia et al., 2004). Neurons co-cultured with DS astrocytes have greater protection against hydrogen peroxide injury than neurons co-cultured with normal astrocytes (Sebastia et al., 2004). Down syndrome astrocytes show a greater antioxidant capacity against hydrogen peroxide than normal astrocytes, and they partially counteract the oxidative vulnerability of trisomic neurons in cultures (Sebastia et al., 2004). In addition, microarray analyses showed that genes overexpressed in DS astrocytes are involved in energy metabolism and oxidative stress (Helguera et al., 2013). Interestingly, in this study, the authors show that when mitochondrial function is restored, there is increased production of reactive oxygen species and cellular damage, suggesting that reduced DS mitochondrial activity is an adaptive response to avoid injury and preserve essential cellular functions.

## DS astrocytes and S100β

S-100β is a protein-enriched in astrocytes implicated in neuronal growth and differentiation, intracellular calcium signaling transduction, and proliferation and morphogenesis of astrocytes (Michetti et al., 2018). Like the SOD gene, the S100β gene is encoded on chromosome 21 (Allore et al., 1988), and overexpression of S100β during development may be associated with neurological abnormalities described in DS (Marks and Allore, 1990). Indeed, the overexpression of S100β has been found in the temporal lobe of fetuses with DS at 18–19 gestational weeks, neonates and infants (Griffin et al., 1989), in the frontal and temporal lobes of adults with DS (Jorgensen et al., 1990; Mito and Becker, 1993; Royston et al., 1999), and the cerebellum of middle-aged and old Ts1Cje mice, a DS model (Creaú et al., 2016). Moreover, an increase in S100β has been observed in AD brains (Jorgensen et al., 1990). In addition, some studies show that overexpression of astrocytic S100β is positively associated with the number of APP overexpressing neurons and in neurites with abnormal growth (Royston et al., 1999), suggesting that overexpression of S100β could be associated with amyloid plaque formation and AD progression in DS (Griffin et al., 1998). In addition, S100β and APP increase astrogliogenesis and reduce neurogenesis (Guidi et al., 2008; Lu et al., 2011; Coronel et al., 2019), contributing to astrocyte dysfunction in DS.

## Astrocytes and AD pathology in DS

The development of AD-type neuropathology in DS individuals has been observed from 12 years old (Wisniewski et al., 1985). However, the contribution of astrocytes in this process is not completely known. Abundant GFAP-positive astrocytes associated with Aβ42 plaques were reported in the frontal cortex of 15 and 29-years-old individuals with DS (Stoltzner et al., 2000). Another study has reported that astrocytes from the hippocampus and cerebral cortex displayed Aβ1–28 and Aβ40 in their cell bodies and in processes that extended to the vicinity of blood vessels (Gyure et al., 2001). The detection of Aβ in astrocytes may reflect either increased synthesis of Aβ by these cells or enhanced clearance of the peptide by astrocytes (Gyure et al., 2001). In addition, an increase in astrocytic apolipoprotein E expression has been reported in the gray matter of the DS frontal cortex from fetal stages to 24-years-old (Arai et al., 1995). In contrast, a decrease of astrocytic apolipoprotein E was reported in the white matter of the same individuals (Arai et al., 1995), suggesting that apolipoprotein E might be produced in astrocytes at the early phase of the pathological process lead to AD in DS people.

Down syndrome astrocytes also show alterations in mitochondrial transmembrane potential, mitochondrial redox activity, mitochondrial morphology, and ATP levels (Busciglio et al., 2002; Coskun and Busciglio, 2012; Helguera et al., 2013). The metabolic alterations in DS astrocytes could directly influence the processing of APP, resulting in increased levels of AβPP and reduced levels of secreted AβPP (AβPPs). This pattern of AβPP processing can be recapitulated in control astrocytes by inhibiting the mitochondrial metabolism, suggesting that DS astrocytes have altered mitochondrial function caused by AβPP overexpression (Busciglio et al., 2002). Interestingly, it has been shown that the survival of DS neurons increases by astrocyte-produced AβPPs, suggesting that AβPPs may be a neuronal survival factor (Busciglio et al., 2002). The mitochondrial dysfunction in DS may lead to increase intracellular expression of Aβ42 and reduced levels of AβPPs, leading to increased neuronal vulnerability (Busciglio et al., 2002). Altered mitochondrial membrane potential, low levels of ATP, and increased reactive oxygen species have also been found in cortical astrocytes obtained from TsCje mice (Shukkur et al., 2006). Furthermore, mitochondrial dysfunction might be associated with an increase in Tau hyperphosphorylation without NFTs formation through an increase in GSK3β (glycogen synthase kinase 3β) and JNK/SAPK (Jun amino-terminal kinase/stress-activated protein kinase) activities (Shukkur et al., 2006).

It has been established that neuroinflammation and glial activation play a significant role in the development and progression of AD (Wilcock, 2012). Astrocytes from the frontal cortex of fetuses, neonates, children, and adults with DS display upregulation of the inflammatory cytokines IL-1 and S100β (Griffin et al., 1989). Further studies have shown that IL-1 regulates the synthesis of AβPP (Batarseh et al., 2016), suggesting that inflammatory processes participate in the pathogenesis of AD in DS (Martini et al., 2022).

## Microglial cells

Microglial cells, the resident macrophages of the CNS, account for 5–12% of the total number of cells in the human brain (Lawson et al., 1990). Microglial cells are derived from yolk sac primitive myeloid progenitors that migrate to the CNS during embryogenesis (Ginhoux et al., 2010). Their brain distribution is heterogeneous, and high microglial cell numbers are observed in specific anatomic regions (Lawson et al., 1990). During brain development, microglial cells are more proliferative than in adult stages and typically display an amoeboid morphology (Perez-Pouchoulen et al., 2015). Such morphology has been associated with its early role in shaping neuronal circuits, tissue remodeling, and cell phagocytosis (Prinz et al., 2019).

In the adult brain and under physiological conditions, microglial cells display a resting or surveying state (Nimmerjahn et al., 2005). Adult microglia are characterized by a small soma size and elongated processes that allow them to constantly probe their surrounding environment and interact with neighboring cells (Nimmerjahn et al., 2005). In response to a harmful stimulus, microglial cells undergo different intermediate stages displaying changes in their morphology, motility, phagocytic status, and gene and protein expression (Prinz et al., 2019). Microglial cells become reactive upon sensing acute injury, retracting their processes, and adopting an amoeboid morphology (Stence et al., 2001). This fully activated state has been associated with microglial phagocytic functions.

## Microglia in DS

Microglial chronic activation is a common feature of neurodegenerative disorders. Such chronic activation interferes with microglial homeostatic functions and contributes to the exacerbated neuroinflammatory profile observed in neurological conditions. In the brains of fetuses with DS (17–22 gestational weeks), microglial cells, including amoeboid and ramified microglia, emerge at the same timepoint as in control brains (Wierzba-Bobrowicz et al., 1999). However, an increase in ramified microglial cells has been reported in the frontal lobe, mesencephalon, and cerebellum from fetuses with DS (Wierzba-Bobrowicz et al., 1999). Further studies have shown that CD68 and HLA-DR positive cells are detectable in the germinal layers from control and DS fetal brains at 14 gestational weeks (Kanaumi et al., 2013). However, an increase in these cells was also observed in DS fetal brains. Furthermore, the increase in CD68+ microglial cells in the germinal matrix is higher in DS than in control brains, suggesting that microglial phagocytic activity might be associated with defects in neurogenesis and apoptosis (Kanaumi et al., 2013). Another study found that microglial cells from fetuses, neonates, children, and adults with DS were immunoreactive to IL-1β, a pro-inflammatory cytokine, suggesting increased neuroinflammation at the fetal stages (Griffin et al., 1989). Similarly, increased production of IL-1β and superoxide anion was reported in microglial cells from a DS mouse model at the fetal stage (embryonic day 15) (Colton et al., 1990, 1991). Interestingly, analysis of cell-free mRNA in the amniotic fluid suggests an increase in oxidative stress signaling pathways in DS fetuses (Slonim et al., 2009). Such an increase may interfere with glial cell modulatory processes and negatively impact brain development in DS. Further research is needed to fully understand the role of microglial and astroglial cells during brain development in DS.

During infancy, morphological analyses have revealed that microglial cells from children with DS displayed an increased soma size and soma size-to-process-length ratio than age-matched controls, suggesting that microglial cells might be reactive (Flores-Aguilar et al., 2020).

## Microglia and Alzheimer's disease in Down syndrome

Microglial activation is also present in adolescents and young adults with DS and becomes exacerbated in adults with overt AD pathology (Stoltzner et al., 2000; Head et al., 2001; Flores-Aguilar et al., 2020; Martini et al., 2020; Palmer et al., 2021). Notably, a transcriptomic signature related to AD and aging has been reported in DS microglial cells before the development of full-blown AD pathology (Palmer et al., 2021). This microglial signature is characterized by an increased expression of C1q-complement-related genes (Palmer et al., 2021). In line with this, an increase in cytokine gene and protein expression has been reported in fetuses, children, and young adults with DS, well-before developing a full-blown AD pathology (Wilcock et al., 2015; Flores-Aguilar et al., 2020). Moreover, the overexpression of IFN-related genes encoded in chromosome 21 might impact microglial reactivity and neuroinflammation in DS (Wilcock and Griffin, 2013). In addition, individuals with DS display a biphasic expression of the triggering receptor expressed on myeloid cells 2 (TREM2) (Raha-Chowdhury et al., 2018; Weber et al., 2020). Triggering receptor expressed on myeloid cells 2 is involved in microglial phagocytosis (Krasemann et al., 2017; Mazaheri et al., 2017), and its serum and plasma levels are increased in young adults with DS (Raha-Chowdhury et al., 2018; Weber et al., 2020). In contrast, downregulation of TREM2 expression has been reported in serum and the frontal cortex of adults with DS (Raha-Chowdhury et al., 2018). Such biphasic expression, also reported in AD (Kleinberger et al., 2014; Suarez-Calvet et al., 2016), might influence microglial activity at different stages of AD in DS, as exemplified in an AD mouse model (Jay et al., 2017). Moreover, older adults with DS display increased CD64 and CD86 (Wilcock et al., 2015). The expression of these markers has been associated with the formation of immune complexes in the brain and subsequent microglial activation (Edwards et al., 2006; Sudduth et al., 2013). Therefore, microglial activation might also be associated with cerebrovascular dysfunction and the extravasation of serum proteins into the brain (Wilcock et al., 2015).

Microglial dystrophy has been reported in older adults with DS (Xue and Streit, 2011; Flores-Aguilar et al., 2020; Martini et al., 2020). Dystrophic microglia in DS brains are characterized by process swelling, bead formation, and cell rupture. Such dystrophic phenotype might be associated with advanced stages of AD neuropathology commonly present in the brains of older adults with DS.

Interestingly, the brains of individuals with DS also display an increase in rod-like microglial cells (Flores-Aguilar et al., 2020; Martini et al., 2020). Higher rod-like microglia counts have been reported in the frontal cortex of young adults and adults with DS and in the posterior cingulate cortex of adults with DS and AD (Flores-Aguilar et al., 2020; Martini et al., 2020). Rod-like microglial cells have also been observed in the CA1 region of the hippocampus in DS brains (Head et al., 2001). Moreover, rod-like microglial cells might align to p-tau positive neurons in the DS frontal cortex (Flores-Aguilar et al., 2020). Rod-like microglial cells have been observed in other neurodegenerative disorders such as AD (Bachstetter et al., 2015) and might be linked to the appearance of tau pathology (Adaikkan et al., 2019; Malcolm et al., 2019). However, their exact function is not well-understood (Giordano et al., 2021).

Studies in DS animal models highlight the critical role that microglial cells exert in DS. Microglial activation has been observed in the Ts65Dn and Dp(16) DS mouse models (Hunter et al., 2004; Lockrow et al., 2011; Rueda et al., 2018; Illouz et al., 2019; Hamlett et al., 2020; Pinto et al., 2020). Microglial depletion in the Dp(16) DS mouse model improves cognition in juvenile mice and neuronal spine and activity (Pinto et al., 2020). Further, anti-inflammatory treatments in DS mouse models promote microglial homeostasis and rescue cognitive deficits (Hamlett et al., 2020; Pinto et al., 2020). Such observations support that microglial chronic activation and neuroinflammation might have a detrimental role in DS. As mentioned above, AD neuropathology might also be involved in the life-long microglial activation observed in DS brains (Flores-Aguilar et al., 2020). DNA vaccination against the Aβ_1−11_ fragment in the Ts65Dn DS mouse model restored microglial homeostasis, rescued cognitive deficits, and reduced neurodegeneration and Aβ levels (Illouz et al., 2019).

## Conclusion

**Figure 1:**
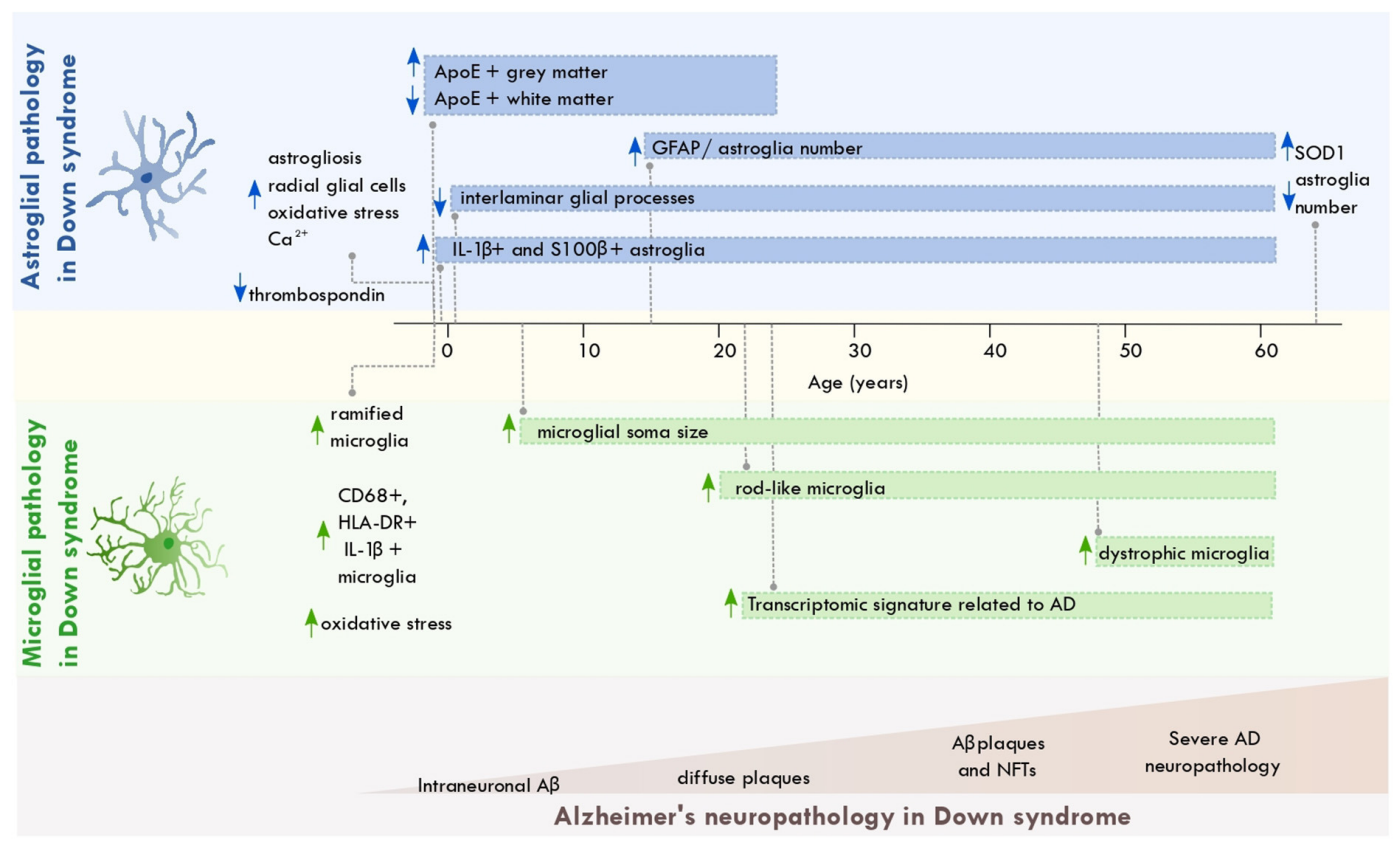
Astroglial and microglial pathology across the lifespan of individuals with Down syndrome. Scheme depicting the most significant changes in astrocytes and microglia from individuals with Down syndrome across aging and along with the progression of AD neuropathology. The brain regions where these disruptions occur are described in the main text. Aβ, amyloid beta; NFT, neurofibrillary tangles; ApoE, apolipoprotein E; AD, Alzheimer's disease; SOD, superoxide dismutase 1; GFAP, glial fibrillary acidic protein.

Astroglial and microglial cells display pathological alterations across the lifespan of individuals with DS. Down syndrome astrocytes show metabolic alterations involving pathways associated with oxidative stress, calcium regulation, and Aβ production. These alterations might result from the trisomic condition that occurs in these cells (Figure 1). Astrocytic dysfunction might interfere with neuronal excitability and the balance of excitatory and inhibitory transmission (Cresto et al., 2019). Such dysfunction could contribute to learning and memory impairments observed in DS (Fernández-Blanco and Dierssen, 2020).

On the other hand, microglial cells display several reactive states across the lifespan of individuals with DS. Such activation states might be associated with AD neuropathology, increased CNS and peripheral inflammation, and the triplication of immune-related genes encoded in chromosome 21 (Wilcock and Griffin, 2013). Given the involvement of microglial cells in the development of AD pathology, chronic microglial activation in DS might feed the progression of AD in this population.

Further studies are needed to fully understand the pathological alterations of glial cells in DS and their contribution to AD pathology. This could lead to identifying potential therapeutics targeting dysfunctional astroglial and microglial cells in DS.

## Author contributions

Both authors listed have made a substantial, direct, and intellectual contribution to the work and approved it for publication.

## Funding

This work was supported by PAPIIT IN-304817 and PAPIME-PE302320 (OG).

## Conflict of interest

The authors declare that the research was conducted in the absence of any commercial or financial relationships that could be construed as a potential conflict of interest.

## Publisher's note

All claims expressed in this article are solely those of the authors and do not necessarily represent those of their affiliated organizations, or those of the publisher, the editors and the reviewers. Any product that may be evaluated in this article, or claim that may be made by its manufacturer, is not guaranteed or endorsed by the publisher.
